# Cardiac Ion Channel Regulation in Obesity and the Metabolic Syndrome: Relevance to Long QT Syndrome and Atrial Fibrillation

**DOI:** 10.3389/fphys.2017.00431

**Published:** 2017-06-21

**Authors:** Ademuyiwa S. Aromolaran, Mohamed Boutjdir

**Affiliations:** ^1^Cardiovascular Research Program, VA New York Harbor Healthcare SystemBrooklyn, NY, United States; ^2^Departments of Medicine, Cell Biology and Pharmacology, State University of New York Downstate Medical CenterBrooklyn, NY, United States; ^3^Department of Medicine, New York University School of MedicineNew York, NY, United States

**Keywords:** high-fat diet, obesity, metabolic syndrome, ion channel remodeling, long QT syndrome, atrial fibrillation

## Abstract

Obesity and its associated metabolic dysregulation leading to metabolic syndrome is an epidemic that poses a significant public health problem. More than one-third of the world population is overweight or obese leading to enhanced risk of cardiovascular disease (CVD) incidence and mortality. Obesity predisposes to atrial fibrillation, ventricular, and supraventricular arrhythmias; conditions that are underlain by dysfunction in electrical activity of the heart. To date, current therapeutic options for cardiomyopathy of obesity are limited, suggesting that there is considerable room for development of therapeutic interventions with novel mechanisms of action that will help normalize rhythm in obese patients. Emerging candidates for modulation by obesity are cardiac ion channels and Ca handling proteins. However, the underlying molecular mechanisms of the impact of obesity on these channels/Ca handling proteins remain incompletely understood. Obesity is marked by accumulation of adipose tissue associated with a variety of adverse adaptations including dyslipidemia (or abnormal levels of serum free fatty acids), increased secretion of pro-inflammatory cytokines, fibrosis, hyperglycemia, and insulin resistance, that will cause electrical remodeling and thus predispose to arrhythmias. Further, adipose tissue is also associated with the accumulation of subcutaneous and visceral fat, which are marked by distinct signaling mechanisms. Thus, there may also be functional differences in the outcome of regional distribution of fat deposits on ion channel/Ca handling proteins expression. Evaluating alterations in their functional expression in obesity will lead to progress in the knowledge about the mechanisms responsible for obesity-related arrhythmias. These advances are likely to reveal new targets for pharmacological modulation. The objective of this article is to review cardiac ion channel/Ca handling proteins remodeling that predispose to arrhythmias. Understanding how obesity and related mechanisms lead to cardiac electrical remodeling is likely to have a significant medical and economic impact.

## Introduction

Obesity is associated with increased accumulation of body fat and significant weight gain leading to the development and prevalence of chronic disorders including dyslipidemias, insulin resistance, and type 2 diabetes (Schulze et al., 2016). Recent estimation by the American Heart Association (AHA), revealed that obesity and related health and emotional distress impacts about 16.9% of children and young adults in the United States, while more than 35% of adults are either overweight or obese (Jensen et al., 2014). Several factors including genetic, environmental, and developmental factors (Leibel, 1997) play a role in the excessive progressive weight gain that leads to obesity. Thus, the development of obesity is dependent on maintaining a healthy energetic balance, largely determined by a multifactorial process including physiological, behavioral, and psychological processes that regulate the delicate balance between food intake and energy expenditure.

The normal metabolic state of the body is maintained by feeding behavior, fat, and glucose metabolism (Pedram and Sun, 2014), and changes in pro-inflammatory cytokines (Guo et al., 2012) including interleukin-1 (IL-1), interleukin-2 (IL-2), interleukin-6 (IL-6), tumor necrosis factor-alpha (TNF-α), and tumor necrosis factor-beta (TGF-β). In obesity, there is marked accumulation of adipose tissue leading to metabolic syndrome which is associated with dyslipidemia (or abnormal levels of serum free fatty acids, FFA), increased secretion of pro-inflammatory cytokines, insulin resistance, hyperglycemia (Sonnenberg et al., 2004), fibrosis (Abed et al., 2013; Ternacle et al., 2017), and hyperuricemia (Viazzi et al., 2017). To date most studies have provided important insights on the impact of individual disorders that contribute to metabolic syndrome on cardiovascular disease (CVD) (Maharani et al., 2016); however, specific cardiac alterations by metabolic syndrome remain poorly understood.

Long QT syndrome (LQTS) is a condition that predisposes patients to an elevated risk for syncope, ventricular arrhythmias and sudden cardiac death. LQTS underlain by congenital mutations in ion channel subunits is somewhat rare and only affect ~1 in 2000 births (Schwartz et al., 2009; Beitland et al., 2014). In most cases life-threatening arrhythmias are triggered during emotional stress (fear, anger, postpartum state, and loud noises), exercise especially during swimming (Splawski et al., 2000), and by obesity and/or obesity-related diseases (Scherer and Hill, 2016). LQTS is also drug-induced and is the more common form (Kannankeril et al., 2010; Beitland et al., 2014). Therefore, it is relevant to determine the impact of the functional interplay between these triggers, and how they affect disease outcomes.

Atrial fibrillation (AF) or rapid and irregular activation of the atrium, is the most common arrhythmia in both males and females (Abed et al., 2013). The incidence of AF is higher in men, but the risks of stroke and AF-related mortality are significantly higher in women (Fang et al., 2005), suggesting sex-related differences in the underlying molecular mechanisms involved in increased AF risks. Obesity is also a key contributor to the expanding prevalence of AF and according to population-based cohort studies, obese individuals have a 49% increased risk of developing AF compared to non-obese individuals (Wanahita et al., 2008). Furthermore, obese men and women showed respectively 71 and 101% increased risk of developing AF and AF-related stroke compared to the non-obese cohorts; yet, there is a paucity of AF studies that have vigorously assessed the role of gender in cardiomyopathies of obesity. Obesity is also associated with an increased prevalence of hypertension, coronary artery disease, and congestive heart failure (Iacobellis et al., 2002); all of which present as risk factors for thromboembolic stroke in patients with AF. Metabolic syndrome in the context of obesity, diabetes, and hypertension remains one of the major public health challenges worldwide (Poirier et al., 2006). According to a recent AHA report, metabolic syndrome affects ~35% of the adult population in the United States (Association, 2016)^1^ suggesting that metabolic syndrome may be a risk factor for onset of AF in patients.

**Figure 1:**
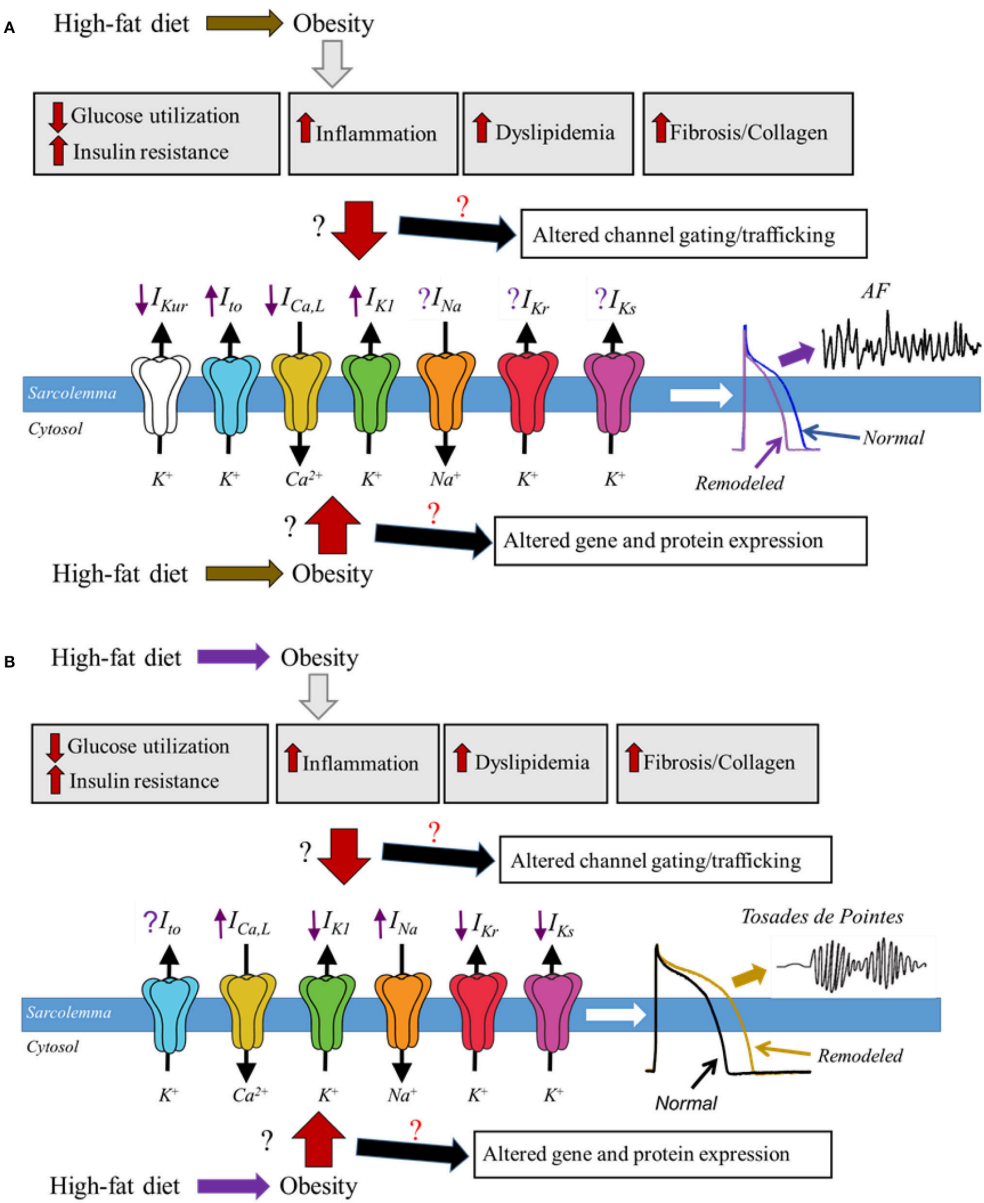
Schematic representation of potential molecular mechanisms of obesity and ion channel remodeling process that may underlie cardiomyopathies of obesity. In diet induced obesity, the associated enlarged adipose tissue leads to altered glucose utilization and insulin resistance, increased levels of proinflammatory cytokines, dyslipidemia, fibrosis, and increased accumulation of collagen; all of which are likely to play a *pivotal* role in remodeling of major atrial and ventricular ion channels leading to either a faster **(A)** or delayed **(B)**, repolarization and predispose obese patients to respectively AF and *Torsades de Pointes*. How and whether obesity molecular mechanisms alter the functional expression of cardiac ion channels is poorly understood, and we have denoted this lack of clarity in this cartoon as the red and black ? We expect that obesity-mediated remodeling process may occur though altered gene and protein expression of ion channel subunits, trafficking and/or gating defects. Distinguishing among these signaling pathways is likely to provide mechanistic insights that will inform on targeted therapy. The purple arrows indicate up-regulation (when pointing upward) or down-regulation (when pointing downward); predicted based on reported functional expression of distinct ion channels in AF and LQTS. The purple ? represents unresolved role of *I*_*Kr*_ and *I*_*Ks*_ in AF and *I*_*to*_ in *Torsades de Pointes*.

The expanding obesity epidemic and the associated increase in CVD underscores the importance in understanding the underlying molecular mechanisms in order to create targeted therapeutic treatments. Because of ethical limitations associated with a vigorous assessment of the mechanisms of human obesity there are limited human studies; however there has been an significant wealth of information about the pathophysiological changes originating from animal models of obesity (Wong et al., 2016). Whether and how the relative functional expression of major cardiac ion channels is altered in obesity remains unknown (Figure 1). However, despite a lack of data, there is increasing indirect evidence for modulation of cardiac ion channel function by distinct obesity-associated factors including dyslipidemia (Aromolaran et al., 2016), leptin (Lin et al., 2013), hyperglycemia (Zhang et al., 2006), and pro-inflammatory cytokines (Zhao et al., 2016). Although these studies have provided crucial mechanistic insights, the increased risks of arrhythmic events in obese patients remain.

Therefore, considering the impact of obesity on life expectancy and the limitations associated with the availability of current treatment options, this review will focus on major atrial and ventricular ion channels/Ca handling proteins and their modulations in obesity (Table 1). Furthermore, the association of obesity with LQTS (Huang et al., 2013) and AF (Abed et al., 2013; Abed and Wittert, 2013; Mahajan et al., 2015), supports the view that common mechanisms may underlie cardiomyopathies of obesity. We also discuss the pathophysiology of cardiac Na, Ca, and K channels and Na/Ca exchanger in the context of LQTS and AF to reveal unacknowledged areas of obesity induced cardiomyopathies that warrant further investigation.

**Table 1:** Altered functional expression of ion channels in animal models of HFD induced obesity.

**Current**	**Gene**	**mRNA**	**Protein**	**Current density**	**Obese model**	**Cardiac tissue**	**QT_c_**	**Reference**
*I_*Na*_*	SCNA5	NR	NR	↔	Rat (SD)	Ventricle	↑	Axelsen et al., 2015
		↑	NR	↑^*^	Rat (WR)	Ventricle	NR	Ashrafi et al., 2016
*I_*Ca, L*_*	CACNA1c	NR	NR	↔	Rat (SDCD)	Ventricle	↑	Ricci et al., 2006
		↔	NR	NR	Rat (WR)	WH	NR	Lima-Leopoldo et al., 2008
		↑	NR	↓	Rat (ZDF)	Ventricle	NR	Howarth et al., 2012
		NR	↔	NR	Rat (WR)	Ventricle	NR	Leopoldo et al., 2011
		NR	↓	↓	Rat (OZR)	Ventricle	↑	Lin et al., 2012
		↓	NR	NR	Rat (WR)	WH	NR	Lima-Leopoldo et al., 2013
		↑	NR	↑^*^	Rat (WR)	Ventricle	NR	Ashrafi et al., 2016
		↓	NR	NR	Gerbils	WH	NR	Sahraoui et al., 2016
		NR	NR	↓	Rabbit	Ventricle	NR	Luo et al., 2004
		NR	↓	↓	Mice (C57BL/6J/*db*/*db*)	Ventricle	NR	Pereira et al., 2006
*I_*to*_*	K_v_4.2/K_v_4.3	NR	NR	↔	Rat (SDCD)	Ventricle	NR	Ricci et al., 2006
		NR	↑	NR	Mice (ICR)	Atria	NR	Ricci et al., 2006
		NR	↔	NR	Mice (C57BL/6J)	Ventricle	↑	Huang et al., 2013
		NR	NR	↔	Rat (SD)	Ventricle	↑	Axelsen et al., 2015
		↑	NR	↑^*^	Rat (WR)	Ventricle	NR	Ashrafi et al., 2016
	K_v_1.4	↑	NR	↑^*^	Rat (WR)	Ventricle	NR	Ashrafi et al., 2016
*I_*Kur*_*	K_v_1.5	↓	↓	NR	Mice (C57BL/6J)	Ventricle	↑	Huang et al., 2013
		↑	NR	↑^*^	Rat (WR)	Ventricle	NR	Ashrafi et al., 2016
		NR	↑	NR	Mice (ICR)	Atria	NR	Ricci et al., 2006
*I_*K*_*	*I_*Kr*_*:ERG	↓	NR	NR	Rat (WR)	Ventricle	NR	Ashrafi et al., 2016
	*I_*Ks*_*:KCNQ1	↑	NR	NR	Rat (WR)	Ventricle	NR	Ashrafi et al., 2016
	*I_*K*_*	NR	NR	↔	Rat (SDCD)	Ventricle	NR	Ricci et al., 2006
		NR	NR	↑	Guinea pig	Atria	NR	Aromolaran et al., 2016
*I_*K1*_*	K_ir_2.1	↑	NR	↑^*^	Rat (WR)	Ventricle	NR	Ashrafi et al., 2016

↑, Increased; ↓, decreased; ↔, no change;

* *predicted from computer simulations; NR, not reported; SD, Sprague Dawley; WR, Wistar Rats; SDCD, Sprague Dawley Cesarean Derived; ZDF, Zucker Diabetic fatty rat; OZR, Obese Zucker Rat; ICR, imprinting control region; QT_c_, QT interval corrected for heart rate; WH, whole heart*.

## Ionic mechanisms of cardiomyopathies of obesity

The physiological link between the cardiac action potential (AP) and its ionic channels/exchanger is vital for mechanistic insights into the clinical consequences that occur when there are disease-induced changes in the functional properties of these ion channels/exchanger. In the human heart, the AP is defined by: membrane depolarization or phase 0 due to a large sodium (Na) current (*I*_*Na*_), through voltage-gated Na channel with subsequent calcium (Ca), entry through voltage-gated L-type (*I*_*Ca, L*_) channels and the sodium-calcium exchanger current (*I*_*NCX*_). When in the forward mode the *I*_*NCX*_ exchanges one Ca ion for three Na ions leading to a net depolarizing inward current (Bers and Despa, 2009). The plateau phase of the AP is maintained by a balance between inward and outward currents; repolarization is controlled by fast transient outward potassium (K) currents (*I*_*to*_), the rapid component of the delayed rectifier K current (*I*_*Kr*_) and the slowly activating component of the delayed rectifier (*I*_*Ks*_). In the atria, repolarization is largely controlled by the ultra-rapid delayed rectifier K current (*I*_*Kur*_) (Tian et al., 2006), and plays a pivotal role in the triangular profile of the atrial action potential (Ford et al., 2013). In both the ventricles and atria, the resting membrane potential is determined by the inwardly rectifying K current (*I*_*K1*_) (Varro et al., 1993). In clinical terms, overall heart electrical activity is defined on surface electrocardiogram (ECG) as P-wave duration and amplitude, P-R interval, QRS complex, and QT interval (Byrnes and Costantini, 2017). Prolongation in corrected QT (QT_c_) interval such as LQTS (QT_c_ intervals > 440–470 ms in men and > 460–480 ms in women) (Schwartz et al., 2009), or an abbreviated QT_c_ or short QT syndrome (SQT; <360 ms in men and <370 ms in women) (Brugada et al., 2004) predispose to arrhythmic events. The pathophysiology of congenital or acquired LQTS is generally defined by a decrease in repolarizing currents (Aromolaran et al., 2014; Puckerin et al., 2016) or an increase in depolarizing currents (Wehrens et al., 2003; Fredj et al., 2006; Cheng et al., 2011; Hsiao et al., 2013). In obese patients cardiomyopathies are manifested as longer P-wave, and increased QT_c_ dispersion (Seyfeli et al., 2006; Nielsen et al., 2013b). Since altered QT_c_ is also associated with sudden cardiac death (SCD), Drenick et al. found that in obesity SCD is 12-fold higher in patients aged 25–34, and 6-fold higher in in age group 35–44 years (Drenick et al., 1980). On the other hand, Kannel et al. found that there was no association between obesity and SCD (Kannel et al., 1998). These opposing conclusions further underscore the complexity of the molecular mechanisms that underlie obesity-related arrhythmias. In the context of AF, altered ion channel function that either increases outward K currents (Aromolaran et al., 2016) or decrease inward Ca currents (Van Wagoner et al., 1997, 1999; Christ et al., 2004; Mancarella et al., 2008) is likely to accelerate repolarization, leading to abbreviated AP duration (APD) (Boutjdir et al., 1986), atrial refractoriness (Boutjdir et al., 1986), and thereby promoting ectopic firing and single/multiple wave re-entrant mechanisms (Wakili et al., 2011; Nattel and Dobrev, 2017). However, there are have been several reports that demonstrate that both prolonged (Pai and Rawles, 1989; Mandyam et al., 2013; Nielsen et al., 2013a), and shortened (Poglajen et al., 2006; Saluja et al., 2008; Nielsen et al., 2013a), QT_c_ are associated with the onset of AF. Collectively these observations suggest that obesity has direct impact on the electrical activity of the heart.

## Molecular changes of cardiac ion channels by obesity

### Depolarizing Na current (*I*_*Na*_)

As pointed out above, the upstroke or initial phase of the cardiac AP is controlled by the entry of *I*_*Na*_ through voltage-gated Na channels, specifically Na_v_1.5, which is encoded by the gene SCNA5 (Lieve et al., 2017). The Na channel is composed of a single pore-forming α-subunit and its regulatory β-subunits (β1-4, encoded by *SCN1B-4B*) that are widely expressed in the heart (Valdivia et al., 2010). Whether and how *I*_*Na*_ is directly modulated in obesity is poorly understood; however, there is indirect evidence for the potential modulation of *I*_*Na*_ in obesity. For example, Lin et al. demonstrated that acute exposure (1-h), of rabbit atrial myocytes to leptin, a peptide hormone involved in regulation of food intake (Wildman et al., 2000) and elevated in obesity (Ravussin et al., 2014), increased peak *I*_*Na*_ density (Lin et al., 2013). Peak *I*_*Na*_ density is also increased by pro-inflammatory cytokines (Zhao et al., 2016), while late *I*_*Na*_ current is increased by FFAs (Lin et al., 2014), in line with altered functional expression of *I*_*Na*_ in obesity.

Changes in serum FFAs are associated with increased risk of cardiac arrhythmias (Aromolaran et al., 2016), and suggests an important role for a functional interplay between cardiac ion channels and FFAs in myocardial disease. The cardiac *I*_*Na*_ is a prime candidate for electrical disturbances caused by exposure of the heart to elevated levels of FFAs. Despite the role of *I*_*Na*_ in normal cardiac depolarization and therefore heart excitability (Luo and Rudy, 1991), there is still controversy about its contribution to metabolic disease associated arrhythmias. Previously, O'Connell et al., demonstrated that while short-term exposure of ovine left atrial myocytes to the saturated FFA, stearic acid (SA) abbreviated the APD, *I*_*Na*_ remained essentially unchanged (O'Connell et al., 2015). In dog ventricular myocytes elevated levels of FFAs increased *I*_*Na*_ density, altered its gating properties, and increased cardiac excitability through augmentation of intracellular Ca concentration (Biet et al., 2014). By contrast, hypercholesterolemic rabbits displayed significantly depressed ventricular peak *I*_*Na*_, leftward shift in the inactivation potential and a slowed time course of recovery when compared to normolipidemic control myocytes (Wu et al., 1997). Considering the implications of altered *I*_*Na*_ electrical remodeling, a good understanding of the molecular mechanisms underlying Na channel gating and functional regulation in obese heart is critical for fundamental insights into the prevalent condition of LQTS and metabolic disease-related arrhythmias in patients.

#### *I*_*Na*_ in LQTS and obesity

Similar to observations in the presence of obesity biomarkers, congenital gain-of-function mutations in Na_v_1.5 channel subunits increases *I*_*Na*_ density and delays ventricular repolarization leading to prolongation of the QT interval in LQT3, and accounts for about 5–10% of genotype-positive patients (Splawski et al., 2000). Furthermore, a rat model of diet-induced obesity (DIO) displayed prolongation of the QRS complex despite unchanged densities of peak *I*_*Na*_, and/or outward K currents measured in ventricular myocytes (Axelsen et al., 2015) (Table 1). These observations emphasize the notion that ion channel functional expression may be regulated differently in obesity and/or metabolic diseases.

#### *I*_*Na*_ in AF and obesity

In the context of AF, increased *I*_*Na*_ density, as seen with the obesity biomarkers, would be surprising considering that *I*_*Na*_ is either unchanged (Bosch et al., 1999), or slightly reduced (Sossalla et al., 2010) in AF patients. There have also been reports of AF-related decreases in Na channel mRNA and protein expression, current density, and atrial conduction delay (Gaspo et al., 1997). These observations raise the possibility that *I*_*Na*_ functional expression may initially increase and then decrease with progressive weight gain/obesity and/or AF progression. Further studies are needed to test this hypothesis and are likely to provide molecular insight as to whether mRNA and protein expression levels and density of *I*_*Na*_ fluctuates with the severity of obesity or AF.

## Voltage-gated L-type Ca channels (*I*_*Ca, L*_), Ca handling proteins and obesity

### L-type Ca channels (*I*_*Ca, L*_)

Ca influx through high voltage-activated Ca channels is an important regulator of cellular excitation-contraction (E-C) coupling (Fu et al., 2014). In myocytes, E-C coupling is established through Ca entry, *I*_*Ca, L*_, through Ca channels which in turn triggers Ca release from intracellular Ca stores (Brandenburg et al., 2016). The α or pore-forming subunit (Ca_v_1.2) of the *I*_*Ca, L*_ channel is encoded by the CACNAC1 gene (Catterall et al., 2003; Fu et al., 2013; Qian et al., 2017). *I*_*Ca, L*_ is modulated by interactions with cytoplasmic regulatory subunits (Ca_v_β1-4, Ca_v_α_2_δ1-4, Ca_v_γ1-8) that play a pivotal role in channel gating properties (Colecraft et al., 2002; Yang et al., 2011), subcellular localization, and surface expression of the α-subunits (Yang and Colecraft, 2013; Tetreault et al., 2016).

In the context of metabolic diseases, altered cholesterol and/or FFA content of membranes will be expected to also affect Ca channel function, possibly through decreased cytosolic Ca levels and impaired cardiac contractility. O'Connell et al. demonstrated that short-term exposure of ovine left atrial myocytes to SA caused a significant reduction of *I*_*Ca, L*_ density (O'Connell et al., 2015), in line with a contribution of FFA-mediated atrial *I*_*Ca, L*_ dysfunction in obesity. In ventricular myocytes isolated from New Zealand white rabbits fed a cholesterol-rich diet for 12 weeks, *I*_*Ca, L*_ density was only slightly increased and not significantly different from normal chow-fed controls (Luo et al., 2004). These observations suggest the possibility that atrial *I*_*Ca, L*_ may be more sensitive to alterations in FFAs *in vitro* compared to *in vivo* effects in animal models involving additive effects of multiple combinations of FFAs.

Further, distinct proinflammatory cytokines that are involved in obesity such as IL-1β, IL-6, and TNF-α have also been shown to alter *I*_*Ca, L*_, density, although these studies have yielded varying results. IL-1β and IL-6 either decreased (El Khoury et al., 2014) or increased (Hagiwara et al., 2007), *I*_*Ca, L*_ amplitude in ventricular myocytes. In contrast, TNF-α decreased *I*_*Ca, L*_ density and the amplitude of Ca transients in rat ventricular myocytes (Duncan et al., 2010), which would be consistent with an abbreviated ventricular APD.

The effects of obesity on the functional expression of *I*_*Ca, L*_ has also been investigated in animal models with contrasting outcomes. For example, in rats fed a high-fat diet for 15 weeks, obesity reduced Ca influx, while gene expression of CACNAC1 was either decreased (Leopoldo et al., 2011) or unchanged (Lima-Leopoldo et al., 2008). However, in another study Leopoldo et al. found that mRNA expression of L-type Ca channel is increased at 30 weeks (Lima-Leopoldo et al., 2013). Ashrafi et al. also reported increased mRNA levels of ventricular *I*_*Ca, L*_ after 8 weeks in high fat diet fed rats; while Leopoldo et al. found no change in protein expression (Leopoldo et al., 2011). In a DIO *Psammomys obesus* Gerbil model, Sahraoui et al. found that mRNA and protein expression levels of CACNAC1 was decreased after 16 weeks (Sahraoui et al., 2016), consistent with a contribution of defective *I*_*Ca, L*_ channel gating/trafficking in obesity.

#### *I*_*Ca, L*_ in LQTS and obesity

Gain-of-function inherited mutations in the α or pore-forming subunit of the Ca channel are also associated with ventricular arrhythmias (Fukuyama et al., 2014). Since IL-1β and IL-6 can increase *I*_*Ca, L*_ density, it is possible that IL-1β and IL-6, through their modulation of *I*_*Ca, L*_ function are prime candidates for the electrical remodeling that predispose obese patients to LQTS. Consequently, it will be interesting to determine how relative changes in these mediators correlate with the functional expression of Ca channels with progressive weight gain in animal models of obesity.

In young type 2 Zucker diabetic fatty (ZDR) rat heart, Howarth et al. found that ventricular expression of CACNAC1 genes are upregulated, *I*_*Ca, L*_ density is reduced, and the rate of channel inactivation is prolonged (Howarth et al., 2012) (Table 1). Reduced ventricular *I*_*Ca, L*_ density, impaired channel inactivation, and decreased protein expression of CACNAC1 have also previously been shown to be prominent mechanisms that predispose the obese Zucker rat (OZR), to QT_c_ prolongation (Lin et al., 2012). This is surprising, considering that decreased *I*_*Ca, L*_ density would be expected to contribute to an abbreviated APD, and therefore a shortened QT_c_. Thus, it is possible that in OZR and ZDR the net effect of Ca_v_1.2 channel modulation is defective inactivation of *I*_*Ca, L*_ leading to LQTS.

#### *I*_*Ca, L*_ in AF and obesity

Altered Ca channel function has been implicated in AF pathogenesis (Van Wagoner et al., 1999; Christ et al., 2004; Mancarella et al., 2008). In chronic AF patients, Bosch et al. demonstrated a 70% reduction in *I*_*Ca, L*_ density, and parallel decreases in mRNA and protein levels of CACNAC1 (Bosch et al., 1999); although there are also reports of unchanged CACNAC1(Schotten et al., 2003). *I*_*Ca, L*_ is also reduced in canine AF studies (Yue et al., 1997). There have also been reports of decreased expression of the regulatory subunits β1, β2a, β2b, and α_2_δ2, which is also likely to contribute to the reduction of *I*_*Ca, L*_ density (Gaborit et al., 2005). In the context of arrhythmias, obesity mechanisms that decrease *I*_*Ca, L*_ are also likely to increase the risk of atrial arrhythmias. However, in isolated human atrial myocytes, insulin, which is generally elevated in obesity and metabolic syndrome, has been shown to increase *I*_*Ca, L*_ and slow its inactivation (Maier et al., 1999), which would be in line with prolongation of APD. In a Ca_v_1.3/α1D knock-out (KO) mouse model of AF, decreased *I*_*Ca, L*_ density was also associated with reduced intracellular Ca transients (Mancarella et al., 2008), demonstrating a pivotal role for altered Ca handling proteins in disease mechanisms that act as substrates for onset of AF.

### Ca handling proteins and obesity

Obesity-related altered functional expression of intracellular Ca release channels, such as ryanodine receptors (RyRs) or inositol triphosphate receptors (IP_3_R), involved in regulating the intracellular Ca concentration are also likely to contribute to the pathogenesis of arrhythmias (Table 2). In this context Dincer et al., previously reported a significantly increased phosphorylation of ventricular RyR type 2 (RyR2) in a dog model of metabolic syndrome while RyR2 mRNA and protein expression remained essentially unchanged (Dincer et al., 2006). Similar results were also observed in obese Gerbils (Sahraoui et al., 2016). However, in rabbits that were fed a cholesterol-rich diet for 12 weeks, mRNA levels of RyR were decreased (Luo et al., 2004). Similar results were also seen in *db*/*db* obese mouse myocytes (Pereira et al., 2006). In rats fed a high-fat diet for 8 weeks, obesity increased mRNA levels of ventricular expression of RyR2 (Ashrafi et al., 2016), while Leopoldo et al. showed increased expression in obese rats at 30 weeks and no change of expression at 15 and 45 weeks (Lima-Leopoldo et al., 2013). These contrasting data further emphasize the inconsistencies between studies and supports the notion that early altered transcript expression may not reflect the impact of long-term obesity.

**Table 2:** Altered functional expression of cardiac Ca handling proteins in obesity.

**Ca handling protein**	**mRNA**	**Protein**	**Animal model**	**Cardiac tissue**	**References**
*SERCA2*	↑	NR	Rat (WR, 15 weeks)	WH	Lima-Leopoldo et al., 2008
	NR	↔	Rat (WR, 15 weeks)	WH	Leopoldo et al., 2011
	↓	NR	Rat (WR, 15 weeks)	WH	Lima-Leopoldo et al., 2013
	↑	NR	Rat (WR, 30 weeks)	WH	Lima-Leopoldo et al., 2013
	↓	NR	Rat (WR, 45 weeks)	WH	Lima-Leopoldo et al., 2013
	↓	↓	Gerbils	WH	Sahraoui et al., 2016
	↓	↓	Rabbit (12 weeks)	Ventricle	Luo et al., 2004
	↑	NR	Rat (WR, 8 weeks)	Ventricle	Ashrafi et al., 2016
*RYR*	↓	NR	Rabbit (12 weeks)	Ventricle	Luo et al., 2004
	NR	↔	Mice (C57BL/6J/*db*/*db*)	Ventricle	Pereira et al., 2006
	↑	NR	Rat (WR, 15 weeks)	WH	Lima-Leopoldo et al., 2008
	↔	NR	Rat (WR, 15 weeks)	WH	Lima-Leopoldo et al., 2013
	↑	NR	Rat (WR, 30 weeks)	WH	Lima-Leopoldo et al., 2013
	↔	NR	Rat (WR, 45 weeks)	WH	Lima-Leopoldo et al., 2013
	↑	NR	Rat (WR, 8 weeks)	Ventricle	Ashrafi et al., 2016
	↔	NR	Gerbils	WH	Sahraoui et al., 2016
*IP_*3*_R*	NR	↔	Mice (C57BL/*ob*/*ob*)	Ventricle	Fauconnier et al., 2005
*PLB*	↑	NR	Rat (WR, 15 weeks)	WH	Lima-Leopoldo et al., 2008
	↓	NR	Rat (WR, 15 weeks)	WH	Lima-Leopoldo et al., 2013
	↑	NR	Rat (WR, 30 weeks)	WH	Lima-Leopoldo et al., 2013
	↔	NR	Rat (WR, 45 weeks)	WH	Lima-Leopoldo et al., 2013
	↔	NR	Gerbils	WH	Sahraoui et al., 2016
*NCX*	↑	NR	Rabbit (12 weeks)	Ventricle	Luo et al., 2004
	↔	NR	Rat (WR, 15 weeks)	WH	Lima-Leopoldo et al., 2008
	↓	NR	Rat (WR, 15 weeks)	WH	Lima-Leopoldo et al., 2013
	↑	NR	Rat (WR, 30 weeks)	WH	Lima-Leopoldo et al., 2013
	↓	NR	Rat (WR, 45 weeks)	WH	Lima-Leopoldo et al., 2013
	↔	NR	Gerbils	WH	Sahraoui et al., 2016

*↑, Increased; ↓, decreased; ↔, no change; NR, not reported; WR, Wistar Rats; WH, whole heart*.

Recently in a mouse model of abnormal cardiac lipid accumulation or cardiac lipid overload, mitochondrial oxidative stress was shown to promote increased sarcoplasmic reticulum (SR) Ca leak by oxidizing RyR2 (Joseph et al., 2016). Increased mRNA expression of the RyR2 has also been shown in a rat model of obesity (Lima-Leopoldo et al., 2008; Leopoldo et al., 2011), and further underscores an important role for RYRs in ventricular arrhythmias associated with metabolic disorders. In a *db*/*db* obese type 2 diabetic mouse model, cardiac abnormalities were associated with reduced SR Ca release and reduced expression of ventricular RyRs (Pereira et al., 2006). These outcomes support the notion that the molecular mechanisms and/or signaling pathways that underlie the contribution of RyR to arrhythmogenesis in models of metabolic disorders may differ depending on the pathology.

The role of IP_3_/IP_3_R in diet related obesity is poorly understood. However, in one study that utilized ventricular cardiomyocytes isolated from an *ob*/*ob* mouse model of obesity and type 2 diabetes, it was demonstrated that insulin increased IP_3_ concentration while the expression of type 1 and type 2 IP_3_R was unaltered compared to wild-type controls (Fauconnier et al., 2005). These results further emphasize the importance of studies that will assess the contribution of RyR/IP_3_R signaling pathways to altered Ca regulation in obesity and metabolic disorders.

The Ca^2+^-ATPase pump (SERCA), its inhibitor phospholamban (PLB) (Hicks et al., 1979; Inui et al., 1986), and the Na-Ca exchanger (NCX) are important for, respectively, the SR Ca re-uptake and Ca extrusion, and have been investigated in obese animal models (Table 2). Ashrafi et al. reported increased mRNA levels of SERCA2a and NCX in high fat diet fed rats (Ashrafi et al., 2016). However, the expression of PLB was not investigated in these studies. In rats fed a high-fat diet for 15 weeks, Leopoldo et al. showed increased mRNA levels of SERCA2a and PLB (Lima-Leopoldo et al., 2008). Ashrafi et al. also reported increased mRNA levels of SERCA2a after 8 weeks in high fat diet fed rats (Ashrafi et al., 2016). While Leopoldo et al., found no change in protein expression after 8 weeks (Leopoldo et al., 2011), several other studies showed increased protein expression consistent with increased SERCA2a activity in ventricular myocytes (Xie et al., 2016). In a DIO *Psammomys obesus* Gerbil model, Sahraoui et al. found that mRNA and protein expression levels of SERCA2a decreased after 16 weeks (Sahraoui et al., 2016). Reduced expression of SERCA2a has also been described in a TG-PPAR-γ mouse model of cardiac lipid overload (Joseph et al., 2016) consistent with a pivotal role of Ca handling defects in obesity and metabolic disorders. In hypercholesterolemic rabbits, mRNA levels of ventricular SERCA2 are significantly lower at 12 weeks when compared to rabbits fed normal chow (Luo et al., 2004). However, the expression of SERCA is increased by FFAs in atrial myocytes (Lin et al., 2014), suggesting that the molecular mechanisms that underlie Ca regulation and predispose to arrhythmias in metabolic disorders may be time- and tissue-dependent.

There have also been contrasting data on the expression of NCX in obesity. mRNA levels of ventricular NCX are either increased at 12 weeks in hypercholesterolemic rabbits (Luo et al., 2004), and unchanged (Lima-Leopoldo et al., 2008), or decreased at 15 weeks (Lima-Leopoldo et al., 2013) but increased at 8 weeks (Ashrafi et al., 2016) and 30 weeks (Lima-Leopoldo et al., 2013) in a rat model of obesity. Furthermore, Ca efflux through NCX was also increased in myocytes isolated from a *db*/*db* model of obese type 2 diabetic mice (Pereira et al., 2006) in line with reduced SR Ca load and contractility in this model. In contrast, in a rat obese model the NCX current, (*I*_*NCX*_) was not significantly different from control non-obese rats (Ricci et al., 2006). These results further support the inconsistencies between studies and provide strong evidence for additional studies in more relevant animal models.

Furthermore, there are also discrepancies associated with modulation of NCX function by individual obesity biomarkers. For example, in adult guinea pig ventricular myocytes, insulin was found to increase *I*_*NCX*_ in both freshly isolated and cultured myocytes (Villa-Abrille et al., 2008). In contrast, Lin et al., demonstrated that leptin treated atrial myocytes cells display reduced Ca transient and SR content largely due to reduced *I*_*NCX*_. These observations further emphasize the modulation of the delicate balance of intracellular Ca homeostasis by obesity.

Taken together, the inconsistencies between the mRNA and protein expression and functional current data further demonstrate the importance of electrophysiological experiments in atrial and ventricular myocytes isolated from DIO models. Thus, further studies will be required to determine the pathophysiology of cardiac Ca channels, its auxiliary subunits, and Ca handling proteins in the settings of obesity and associated metabolic syndrome.

## Voltage gated K channels and obesity

### Transient repolarization currents (*I*_*to, fast*_ and *I*_*to, Slow*_)

The transient outward K current, *I*_*to*_ is also an important contributor to cardiac AP waveform, and contributes prominently to the initial and early repolarization phase of atria (Workman et al., 2001; Virag et al., 2011) and ventricular AP (Rosati et al., 2001). In heart, *I*_*to*_ expression has been shown to be greater in the atria when compared to ventricular myocytes, which is likely to underlie the abbreviated atrial APD (Calloe et al., 2011). *I*_*to*_ is defined by two distinct components namely *I*_*to*, *fast*_ and *I*_*to, slow*_ (Patel and Campbell, 2005). *I*_*to*, *fast*_ is generated by a combination of K_v_4.2 and K_v_4.3 channels, which are encoded by *KCND2* and *KCND3* genes, respectively. The auxiliary and regulatory subunits, KChIP2 and dipeptidyl-aminopeptidase-like protein 6 (DPP6), when co-assembled with K_v_4.3, modulates its trafficking and gating properties (Radicke et al., 2005), to generate currents that closely resemble *I*_*to, fast*_.

The slow component of the transient outward current, referred to as *I*_*to, slow*_, is conducted by the voltage gated K channel, K_v_1.4, in the heart (Walsh et al., 2001; Akar et al., 2004; Patel and Campbell, 2005). The K_v_1.4 subunit is encoded by the *KCNA4* gene, and in contrast to *I*_*to, fast*_, it is marked by a fast activation, slower inactivation and slower recovery from inactivation. A transgenic mouse model lacking *I*_*to, fast*_ and *I*_*to, slow*_ displayed reduced *I*_*to*_ density, action potential prolongation and ventricular tachycardia (Guo et al., 2000), consistent with a fundamental contribution of *I*_*to*_ to arrhythmias. Transgenic lipotoxic models, such as the MHC-FATP mouse, also displays reduced *I*_*to, fast*_ density (Marionneau et al., 2008).

#### *I*_*to*_ in LQTS and obesity

In terms of ventricular arrhythmias, decreases in *I*_*to*_ would be expected to delay repolarization and prolong APD, making dysregulation of *I*_*to*_ a plausible contributor to LQTS in obese patients (Grandinetti et al., 2010). The pro-inflammatory cytokine TNF-α has also been shown to depress *I*_*to*_ channel function in ventricular myocytes (Grandy and Fiset, 2009), while the mRNA and/or protein expression of K_v_4.2/K_v_4.3 subunits remained essentially unchanged. The impact of TNFα-mediated *I*_*to*_ reduction on APD has yielded contrasting results, with one report showing no effect (Fernandez-Velasco et al., 2007), and another a prolongation (Grandy and Fiset, 2009), suggesting the possibility of reciprocal regulation of other ventricular ion channels. For example, previous reports have also shown that TNF-α reduces *I*_*Ca, L*_ in myocytes (Duncan et al., 2010), which could in principle offset the depression of *I*_*to*_ and preserve APD. Nonetheless, the data is consistent with a potential contribution of TNF-α to the delayed cardiac repolarization phenotype observed in obese patients (Seyfeli et al., 2006). More TNF-α studies that assess the modulation of major ventricular ion channels are likely to provide a clearer understanding of the role of cytokines on cardiac K channel function and ventricular arrhythmias. Further, previous reports in mouse models of diabetes and obesity have also provided distinct outcomes. There are reports of decreased K_v_4.3 currents with a prolongation of APD in diabetic mice (Shimoni et al., 2004), or unchanged protein levels of K_v_1.4 and K_v_4.2 subunits in a DIO mice despite APD prolongation (Huang et al., 2013), suggesting that the sensitivity of *I*_*to*_ channel function to metabolic disorders and the development of LQTS may vary depending on the underlying pathology.

#### *I*_*to*_ in AF and obesity

Despite a pivotal role of *I*_*to*_ in cardiac repolarization, its contribution to AP prolongation and atrial arrhythmias is poorly understood. In relation to obesity and atrial electrical activity, O'Connell et al. previously demonstrated that saturated FFAs reduced *I*_*to*_ density despite abbreviated APD (O'Connell et al., 2015), emphasizing the complexity of atrial ion channel regulation in diseased hearts. *I*_*to*_ is known to interact with and modulate the activity of other major atrial ion channels including *I*_*Ca, L*_ and the delayed rectifier K channels (Oudit et al., 2001), which may limit its role in AF. Recently, Zhang et al. reported increased atrial electrical activity and an upregulation of K_v_4.3 protein expression in a DIO mouse model (Zhang et al., 2016), but the electrophysiology of K_v_4.3 relative to other atrial and ventricular ionic channels were not assessed in these studies. In addition, an obese rat model which was developed over 14-weeks, ventricular *I*_*to*_, *I*_*Ca, L*_, and *I*_*K*_ densities were not altered (Table 2), when compared to non-obese hearts and further illustrates the complexity of the functional interplay between progressive weight gain, and altered functional expression of cardiac ion channels (Ricci et al., 2006).

## Delayed rectifier K currents (*I*_*Kur*_, *I*_*Kr*_ and *I*_*Ks*_,) and obesity

### Ultra-rapid delayed rectifier K current (*I*_*Kur*_)

The atrial specific *I*_*Kur*_ (or K_v_1.5 encoded by KCNA5), is marked by a fast activation, outward rectification, and a relatively slow inactivation (Bhuyan and Seal, 2017). K_v_1.5 channel subunits exist in macromolecular complexes with its auxiliary β-subunit (K_v_β1.2) which is crucial for its gating and trafficking properties and also its sensitivity to metabolic disorders (Tipparaju et al., 2012). The co-assembly of K_v_1.5 with K_v_β1.2 subunits has also been shown to underlie *I*_*Kur*_ current in the human atrium (Christophersen et al., 2013). Pro-inflammatory cytokine studies have provided some evidence for a potential contribution of atrial K_v_1.5 function in obesity. *I*_*Kur*_ density was shown to be decreased in myocytes isolated from mice treated with TNFα, although mRNA and protein expression levels of *I*_*Kur*_ channel subunits were not altered (Grandy and Fiset, 2009), consistent with TNFα-mediated gating and/or trafficking defects of *I*_*Kur*_ subunits.

#### *I*_*Kur*_ in LQTS and obesity

K_v_1.5 is also a major repolarizing mechanism in mouse ventricular myocyte (Huang et al., 2013), which has allowed evaluation of the impact of obesity on ventricular arrhythmias and the functional role of K_v_1.5. Previously, transgenic lipotoxic models such as the PPARα overexpression mouse marked by prolonged QRS/QT intervals and development of spontaneous ventricular arrhythmias have been shown to display reduced K_v_1.5 currents (Morrow et al., 2011). Recently, Huang et al. using DIO mice, also demonstrated impaired ventricular repolarization and a prolongation of QT interval, which was associated with reduced mRNA and protein levels of K_v_1.5 channel subunits (Huang et al., 2013). The lack of K_v_1.5 electrophysiology in these studies limits a definitive role for K_v_1.5 in LQTS.

#### *I*_*Kur*_ in AF and obesity

The physiological relevance of *I*_*Kur*_ is underscored by data showing that congenital mutations in K_v_1.5 channel subunits increase *I*_*Kur*_ density and shorten atrial APD a condition that predisposes to AF (Christophersen et al., 2013). Similarly, Zhang et al. reported a shortened P-R interval and increased atrial K_v_1.5 protein expression in mice exposed to a high-fat diet for 8-weeks, although these data were not correlated with functional K_v_1.5 channel data nor was AF induced in these studies (Zhang et al., 2016). By comparison, in AF patients *I*_*Kur*_ current density (Van Wagoner et al., 1997), mRNA (Lai et al., 1999), and protein expression levels (Brundel et al., 2001), of K_v_1.5 are decreased. Therefore, these observations would suggest in the DIO mice utilized by Zhang et al. the functional expression of K_v_1.5 may decrease with severity of obesity leading to AF induction. Further, the selective localization and/or expression of K_v_1.5 subunits in atria, and its therapeutic potential demonstrate the need to further elucidate molecular and electrophysiological mechanisms regarding the relative significance and/or contribution of *I*_*Kur*_ to the onset and/or progression of AF in obese patients.

Hyperuricemia has been reported to be associated with obesity, metabolic syndrome, and increased AF risks in patients (Kuwabara et al., 2017). Yet the underlying molecular mechanisms remain unknown. Recently in mouse atrial myocytes uric acid enhanced the protein expression of K_v_1.5 channel leading to an increase in *I*_*Kur*_ density (Maharani et al., 2015). This finding also identifies hyperuricemia as an important contributor to atrial arrhythmias in metabolic disease patients. Therefore, monitoring of the serum urate level could be useful in predictions of the likelihood of AF onset. Controlling the serum urate level in patients might be an important therapeutic option by helping to normalize K_v_1.5 channel expression and therefore sinus rhythm.

There are inconsistencies regarding the effects of obesity on K_v_1.5 in atria and ventricle. For example, reduced functional expression of K_v_1.5 is generally associated with AF (Nunez et al., 2006), and not ventricular arrhythmias, which we expect will also be the case in obese patients with AF. Furthermore, it is important to further assess the role of tissue-specific modifications of obesity-mediated effects on the functional expression K_v_1.5. The human and murine ventricular action potential is defined by different ionic mechanisms suggesting that regulation of sinus rhythm may also be different. Because *I*_*Kur*_ is not expressed in human ventricular myocytes (Nerbonne and Kass, 2005; Ford et al., 2013; Bhuyan and Seal, 2017), *I*_*Kur*_ channels might represent an important therapeutic target for the treatment of atrial arrhythmias without confounding off-target effects on cardiac function. Moreover, the relevance of the contribution of *I*_*Kur*_ to ventricular repolarization in transgenic lipotoxic mouse models needs clarification and warrants further investigation. Therefore, studies utilizing animal models such as obese guinea pig atrial and ventricular myocytes to distinguish among K_v_1.5 functional properties are likely to provide molecular insights that will be readily translatable to common mechanisms in obese human heart and inform on targeted therapeutic interventions.

### The slow and rapid component of the delayed rectifier K current *I*_*k*_ and obesity

The cardiac delayed rectifier K current, or *I*_*K*_ composed of *I*_*Kr*_ and *I*_*Ks*_, is an important regulator of repolarization (Sanguinetti and Jurkiewicz, 1990). In the human heart, *I*_*Kr*_ exists as a tetramer composed of the human ether-á-go-go-related gene (or hERG), 1a and 1b pore-forming or α subunits (Puckerin et al., 2016). There have also been reports that *I*_*Kr*_ is generated by a combination of hERG and the MinK-related peptide 1 protein (Abbott et al., 1999) suggesting that the precise molecular composition of cardiac *I*_*Kr*_ is still a matter of debate. *I*_*Ks*_ is mediated by heteromeric channel complexes composed of pore-forming KCNQ1 (K_v_7.1) subunits and the auxiliary regulatory KCNE1 subunits (Haitin et al., 2008). Channels that conduct *I*_*Kr*_ are fast activating (Sanguinetti and Jurkiewicz, 1991) and *I*_*Ks*_ channels are marked by slowly activating and inactivating kinetics (Aromolaran et al., 2014), and thus underlie their important contribution to repolarization in the late stages of the cardiac AP.

While there have been some studies on the electrophysiological effects of obesity-related molecular processes on some cardiac voltage-gated channels (O'Connell et al., 2015; Aromolaran et al., 2016), there is still a lack of studies that have assessed the functional properties of *I*_*K*_, *I*_*Kr*_**, and *I*_*Ks*_ in obese animal models, most likely due to the lack of expression of these channels in commonly used rodent models (Killeen et al., 2008; Aromolaran et al., 2014).

Previously we have demonstrated that male and female guinea pigs showed significant weight gain and elevated levels of total cholesterol and triglycerides, typically associated with significant weight gain and/or obesity within 50 days on a high-fat diet (HFD) (Aromolaran et al., 2016). Therefore, perturbations including hyperlipidemia (Altarejos et al., 2005), that alter cholesterol levels is likely to have significant implications on the function of ion channels in the lipid bilayer. We also found that obese atrial myocytes displayed an abbreviated APD, and had a significantly larger *I*_*K*_ density compared to the low-fat diet controls. A similar picture was revealed with acute exposure of atrial myocytes to the saturated FFA, palmitic acid (PA), which also increased *I*_*Kr*_ and *I*_*Ks*_ densities in human embryonic kidney 293 (HEK293) cells, demonstrating that removal of PA from HFD is likely to prevent arrhythmic events in obese patients. Compared to PA, the unsaturated FFA, oleic acid (OA), prolonged atrial APD, depressed *I*_*Kr*_ density, and minimally increased *I*_*Ks*_ in HEK293 cells suggesting that increasing OA may prevent atrial arrhythmias in obese patients. The implication of these observations in ventricles is currently unknown; nevertheless, a previous report by Haim et al. have shown that palmitate reduced cardiac contractility, shortened APD, and increased the density of voltage-gated K channels in mouse ventricular myocytes (Haim et al., 2010). Therefore, further studies are required to confirm these in relevant small animal (such as the guinea pig), obese models and to elucidate the significance of these changes to targeted therapeutic interventions in obese patients that present with arrhythmic events.

In a DIO rat model mRNA expression of ERG subunit is significantly reduced (Ashrafi et al., 2016), suggesting that ERG/*I*_*Kr*_ functional expression is altered in obesity, and therefore may contribute to the LQT phenotype seen in clinically obese patients. Since *I*_*Kr*_ does not contribute prominently to repolarization in rat ventricular myocytes (Pond et al., 2000; Aromolaran et al., 2014), vigorous studies in relevant animal models of metabolic disorders utilizing protein and electrophysiological assays will be required to fully understand the role of *I*_*Kr*_ in DIO-related arrhythmias. In this context, Caillier et al. previously showed that guinea pigs with metabolic syndrome displayed prolonged APD in response to the drugs dofetilide and chromanol 293B which are known blockers of *I*_*Kr*_ and *I*_*Ks*_ respectively (Caillier et al., 2012). Furthermore, a recent report by Kannankeril et al. also demonstrated that obese patients display a higher susceptibility to drug-induced QT prolongation mediated by an *I*_*Kr*_ blocker Ibutilide (Kannankeril et al., 2011). These observations suggest that: (1) ventricular *I*_*K*_ is modulated in obesity and these effects are further exacerbated by drugs, which may lead to increased likelihood of fatal arrhythmias; (2) demonstrate the relevance of guinea pig as an model to investigate the pathogenesis of obesity-induced arrhythmias.

*I*_*Kr*_ is also reduced in diabetes (Zhang et al., 2011), which may contribute to lethal ventricular arrhythmias and sudden cardiac death in diabetic patients (Eranti et al., 2016). Hyperglycemia (Gateva et al., 2017), and diabetes (de Simone et al., 2017) are also highly associated with obesity. However, it is currently unknown whether the molecular changes of cardiac ion channels in diabetic patients without obesity recapitulate that in obesity. Therefore, identification of unique pathways that will distinguish between these disease mechanisms and the impact on ion channel expression is likely to have significant implications for novel therapies for arrhythmias associated with metabolic disorders.

Altered levels of proinflammatory cytokines are also associated with prolongation of QT interval in patients with inflammatory diseases (Adlan et al., 2015; Lazzerini et al., 2017), suggesting that cytokines may negatively regulate ion channel function, normal sinus rhythm, and predispose to arrhythmias. Obesity is also associated with elevated levels of inflammatory cytokines (Schmidt et al., 2015), which may also contribute to cardiomyopathies of obesity. Wang et al. previously demonstrated that TNF-α decreased hERG current density in HEK293 cells, significantly depressed *I*_*Kr*_ density and prolonged APD in canine ventricular myocytes, primarily due to changes in reactive oxygen species (ROS) (Wang et al., 2004). Obesity associated hyperglycemia has also been shown to reduced surface expression of hERG channel subunits and hERG1/*I*_*Kr*_ density which is rescued by insulin (Zhang et al., 2006), suggesting that ROS is an important regulator of hERG1/*I*_*Kr*_ functional expression. Recent reports have also demonstrated that the expression of the chaperone protein, heat shock protein 90 (Hsp90) plays a role in ERG channel trafficking defects seen in hyperglycemia (Shi et al., 2015), suggesting that in patients altered expression of ROS and Hsp90 may contribute to cardiomyopathies of obesity. Despite these results whether and how ROS influences Hsp90 function in metabolic disorders is poorly understood and therefore needs further investigation.

#### *I_*K*_* in LQTS and obesity

The functional contribution of *I*_*K*_ to arrhythmias related to metabolic disorders is poorly understood. LQT2-causing hERG 1a mutations account for ~30% of the reported cases of congenital LQTS (Crotti et al., 2008). During emotional stress, there is increased sympathetic stimulation of the heart rate (Schwartz et al., 2001), and up-regulation of *I*_*Kr*_ to shorten APD and normalize cardiac rhythm. As with *I*_*Kr*_, decreases in cardiac *I*_*Ks*_ delays repolarization, and prolongs cardiac APD resulting in LQTS, (Schwartz, 2001). Pathological decreases in *I*_*Ks*_ are generally mediated by congenital mutations in KCNQ1 (LQT1) or KCNE1 (LQT5), with loss-of-function mutations in KCNQ1 accounting for ~30–45% of all inherited LQT cases (Tester et al., 2005). Gating defects, impaired assembly and reduced trafficking are some of the molecular mechanisms that have been proposed to underlie decreased cardiac *I*_*Kr*_ and *I*_*Ks*_ density commonly associated with LQTS (Aromolaran et al., 2014; Puckerin et al., 2016). It will also be important to assess whether and how these molecular processes are altered in obesity.

#### *I_*K*_* in AF and obesity

There is currently limited data on the functional outcomes of *I*_*Kr*_ and *I*_*Ks*_ in non-obese and obese patients with AF. Moreover, previous biochemical studies have also provided conflicting results with some reports showing either no change (Brundel et al., 2001) or decreased (Brundel et al., 2001; Gaborit et al., 2005) mRNA of hERG subunits. *I*_*Ks*_ channel subunit transcripts are also either decreased (Lai et al., 1999) or increased (Gao et al., 2013) in AF patients. How these inconsistent molecular outcomes translate to functional electrophysiological data in AF patients or contribute to onset of AF in obese patients is poorly understood. The lack of clarity is primarily due to difficulties measuring *I*_*Kr*_ and *I*_*Ks*_ in myocytes isolated from AF patients, and warrants a comprehensive assessment of *I*_*K*_ functional expression in small animal models of AF. To this end we are currently assessing the correlation between progressive weight gain and/or obesity and inducibility of arrhythmias including ventricular tachycardia and AF, by means of our previously developed HFD obese male and female guinea pig model (Aromolaran et al., 2016).

## The inwardly rectifying K current (*I_*K1*_*) and obesity

In the heart, non-voltage-gated inwardly rectifying K current (or *I*_*K1*_) is mediated by channel subunits composed of K_ir_2.1, encoded by KCNJ2 (Seyler et al., 2017). *I*_*K1*_ plays a pivotal role in the final stages of the action potential, where its functional expression determines the resting membrane potential (Mancarella et al., 2008). Loss-of-function mutations in the K_ir_2.1 channel subunit that impair trafficking (Barajas-Martinez et al., 2011), and therefore surface expression, depress *I*_*K1*_ density (Tristani-Firouzi et al., 2002), and prolong QT interval leading to LQT7 (Zhang et al., 2005; Leong et al., 2013). There are also reports of increased density of *I*_*K1*_ in ventricular myocytes isolated from MHC-PPARα, a mouse model of cardiac lipid overload, compared to wild-type, but there was no impact on AP waveform due to relative changes in other major ventricular K currents (Marionneau et al., 2008).

We recently reported that atrial myocytes isolated from obese female guinea pigs displayed a more depolarized resting membrane potential, compared to the low-fat diet controls, and obese male guinea pigs (Aromolaran et al., 2016), demonstrating that *I*_*K1*_ density may be reduced in HFD obese female guinea pigs. Moreover, there have also been reports of either unchanged or reduced functional expression of *I*_*K1*_ in AF patients (Li et al., 2000). These inconsistent molecular outcomes of *I*_*K1*_ further emphasize the notion that the molecular mechanisms of AF may differ depending on the underlying pathophysiology. With respect to ventricular arrhythmias, Ashrafi et al. demonstrated significantly increased mRNA levels of K_ir_2.1 channel subunits and *I*_*K1*_ current density in a HFD obese rat model, although these changes had minimal impact on ventricular APD (Ashrafi et al., 2016).

## Discussion

### Strengths of this review

Similar to other reviews (Nerbonne and Kass, 2005; Schmitt et al., 2014; Chen-Izu et al., 2015; Grandi et al., 2017) we have emphasized the important role of ion channels and Ca handling proteins in the pathogenesis of arrhythmias but more importantly to obesity and metabolic syndrome. Due to the contribution of obesity and metabolic disorders to the increasing prevalence of arrhythmias, this review uniquely discusses the latest knowledge related to the molecular mechanisms of how obesity may contribute to the electrical remodeling that underlie arrhythmias such as LQTS and AF. This review also highlights the contribution of altered functional expression of *I*_*K*_ to the remodeling of electrical activity in male and female HFD-induced obese adult guinea pigs and establishes the emerging role of guinea pigs as an important pre-clinical model (Aromolaran et al., 2016). Finally, we have confirmed the urgent need for more translational studies in obese patients, which may reveal key ionic mechanisms that may be targeted for therapeutic interventions and help reduce arrhythmias.

### Limitations of this review

For the purpose of this review article, we used pubmed central, embase, and google scholar databases to search for studies published in English language. Searches were not limited by date restrictions. Searches were free texts and included the following keywords: “high fat diet,” “ion channel,” “obesity,” “metabolic syndrome,” “LQTS,” “atrial fibrillation,” “dyslipidemia,” “cardiac calcium channel,” “cardiac potassium channel,” “cardiac sodium channel,” “Ca handling proteins,” “NCX,” “pro-inflammatory cytokines,” “insulin,” “leptin,” “hyperglycemia,” and “hyperuricemia.” Despite our thorough search, it is also possible that we may have missed relevant studies including non–English language studies. While we have comprehensively focused on the role of voltage gated ion channels and Ca handling proteins as the molecular basis of arrhythmias, there are other entities such as cardiac gap junctions and connexins that have been shown to be altered in obesity (Axelsen et al., 2015; Takahashi et al., 2016) and may also contribute to cardiomyopathies of metabolic disorders. However, the paucity of such studies makes it impossible to draw reliable conclusions and therefore were not discussed in this review.

Since this review describes altered electrical remodeling following short-term high-fat diet and progressive weight gain and/or obesity and not long-term obesity our analysis of the impact of electrical remodeling as an important contributor to cardiomyopathies should be interpreted with caution. Our interpretation is that early relative functional identity of distinct ionic channels and Ca handling proteins may change with severity of obesity. There has also been a paucity of human studies regarding the role of ion channels and Ca handling proteins in the development of obesity-induced arrhythmias; however we have highlighted relevant ones here. While the study is not a comprehensive review of the animal models of obesity or arrhythmias, we discuss the important animal models in the literature and highlight the ability of guinea pigs to be a more appropriate pre-clinical model.

### Future directions

Studies addressing the molecular and functional basis of arrhythmogenesis in relevant animal models of metabolic disorders are just emerging and significant gaps in knowledge remain, warranting further research investigations. Despite these advances, it is still not clear whether common mechanisms underlie altered regulation of ion channels and Ca handling proteins due to inherited mutations or acquired in obesity, diabetes, metabolic syndrome and predispose to fatal arrhythmias. Furthermore, whether weight loss and comorbid management can modulate ion channel expression and function in ways that will help to improve CVD outcomes is poorly understood. Recently, exercise training coupled with caloric restriction has been shown to prevent cardiac dysfunction in obese rats through modulation of Ca handling proteins (Paulino et al., 2010). However, these exciting results in animal studies have not translated into positive results in human clinical trials suggesting that there is considerable room for advances in targeted treatment approaches. One reason for the failure to translate to pre-clinical studies could be due to a paucity of metabolic studies utilizing human myocytes. Therefore, the effort to develop more realistic pre-clinical models that avoid the use of non-human tissues and which instead incorporates cardiomyocytes derived directly from human stem cells from obese patients is likely to provide crucial insight that will advance our knowledge of the association between metabolic disorders and arrhythmias. Although, to our knowledge, there have been no studies using human stem cells as a research paradigm for studying ion channel remodeling in metabolic diseases. Human stem cells, both embryonic stem cells and induced pluripotent, have been successfully used as models of arrhythmias (Priori et al., 2013; Moreau et al., 2017), suggesting that human stem cells could be a realistic pre-clinical human model for studies focused on arrhythmias underlain by metabolic disorders.

## Conclusion

Cardiac arrhythmias, underlain by metabolic disorders, are a pervasive condition that is rapidly becoming an expanding epidemic. Furthermore, current treatment options (β-blockers, catheter ablation, cardioversion, and class I and III antiarrhythmic drugs) (Abed and Wittert, 2013) do not correct the underlying electrical dysfunction and all have significant limitations. In this context, routine electrocardiographic monitoring in obese patients is likely to provide indications of the onset of electrical remodeling which could represent cornerstones for the prevention of arrhythmias. Furthermore, dietary interventions such as removal of saturated FFAs (PA) or the addition of unsaturated FFAs (OA) (Aromolaran et al., 2016) coupled with increased exercise training may also help lower the risk of CVD and arrhythmias. Therefore, bridging the gap between the results obtained using the current pre-clinical models with those obtained thus far in humans will be necessary if we are to find effective strategies that will improve CVD outcomes and enhance the health of all individuals so that they can live longer and more fulfilling lives.

## Author contributions

AA and MB obtained funding, conceived of, and wrote the manuscript.

### Conflict of interest statement

The authors declare that the research was conducted in the absence of any commercial or financial relationships that could be construed as a potential conflict of interest.

## Footnotes

^1^Cardiovascular conditions:What is metabolic syndrome? Available from: https://www.heart.org/idc/groups/heart-public/@wcm/@hcm/documents/downloadable/ucm_300322.pdf.

## Abbreviations

CVD: Cardiovascular disease
DIO: Diet-induced obesity
HFD: High-fat diet
AF: Atrial fibrillation
LQTS: Long QT syndrome
QT_c_: QT interval corrected for heart rate
ECG: Surface electrocardiogram
AP: Action potential
APD: Action potential duration
*I*_*Kr*_: Rapidly activating delayed rectifier K current
*I*_*Ks*_: Slowly activating delayed rectifier K current
*I*_*Na*_: Sodium current
*I*_*Ca*_,_*L*_: L-type Ca current
*I*_*to*_: Fast transient outward K current
*I*_*Kur*_: Ultra-rapid delayed rectifier K current
*I*_*K1*_: Inwardly rectifying K current
*I*_*NCX*_: Sodium-calcium exchanger current
hERG: Human ether-á-go-go-related gene
ERG: Ether-á-go-go-related gene
HEK: Human Embryonic Kidney
NCX: Sodium-calcium exchanger
FFAs: Free-fatty acids
RyR: Ryanodine receptors
IP_3_R: Inositol trisphosphate
SR: Sarcoplasmic reticulum
SERCA: Sarcoplasmic endoplasmic reticulum Ca-ATPase pump
PLB: Phospholamban
SA: Stearic acid
PA: Palmitic acid
OA: Oleic acid
ROS: Reactive oxygen species.
